# The Refuse Disposal of London

**Published:** 1921-04-09

**Authors:** 


### THE REFUSE DISPOSAL OF LONDON.

Before the outbreak of war a conference was called of representatives of Metropolitan Borough Councils to consider and report upon the best means of disposing of the house refuse collected within the county of London in the most sanitary and economical manner. The tonnage to be dealt with is approximately 1,500,000 tons a year, or, say, 5,000 tons per working day. At the present time the greater part is disposed of either by barge or rail. Of the remainder, part is burnt in destructors and part by grinding or crushing and by salvage plants.

The Conference appointed a Committee last July to consider the question, and they have issued an interim report. They state that since the revival of the Conference at the termination of the war some municipal authorities, both provincial and Metropolitan, profiting by the experience gained during the war, have further developed the various schemes inaugurated during that period for salving and selling the usable constituents of the refuse, and the treatment of the remainder for more ready and economical disposal. These schemes are, of necessity, still experimental, and some little time must elapse before their value as a means of solving the very important question of refuse disposal can be fairly estimated.

The Committee have visited several plants where refuse is being dealt with by methods calculated to minimise the cost of disposal, and in some instances actually to become an asset to the rates. During the present period of financial distress, of heavy wages, and of increasing cost of transport, whether by rail, road, or water, the aspect of practicability from the financial point of view (which side of the question has become almost, if not quite, as important as the public health side) must be considered.

### The Rise in Barging Charges.

One example in which the cost of barging and dumping London refuse has increased of late years, which, presumably, is indicative of the circumstances throughout London, is given. At the commencement of the war the rate for barging refuse in the district in question was 2s. 6d. a ton, which, on an annual tonnage of 110,000, gave the sum of £13,750 per annum. To-day the average rate for barging refuse in the same district is 9s. per ton, which, on the same annual tonnage, brings the cost of barging to £49,500 per annum, an increase of £35,750. These figures permit a rough estimate to be formed of the enormous cost to the ratepayers of London of disposing, after collection, of their refuse by the system of barge to dump.

The Committee hope to be able to demonstrate that the system of barging or railing to a shoot can to a large extent be superseded by less costly and much more sanitary methods, but should this hope not be justified, it will be necessary for the Committee to give careful and serious consideration to one of the suggestions put forward by the Deptford Borough Council, that the Borough Councils should buy and work their own barges.

The only instance before them where this is already in operation is that of St. Marylebone Borough Council, who own their own barges and tugs. At the same time, it will be readily realised that year by year the question of finding suitable sites for shoots or dumps becomes more difficult.

Nearly all rural authorities strongly object to land in their areas being used for this purpose, chiefly owing to the fact that many of the dumps are not properly covered over and the land made available for immediate cultivation. There is also the liability to nuisance being caused by fermentations and flies.

### Parliamentary Powers.

Before prominence is given to the salving and treatment of refuse it is suggested that boroughs similarly situated as to locality and conditions might find it advisable to combine, and it is further suggested that Parliamentary powers should be sought for the compulsory acquisition of land for shoots.

Whilst it would, perhaps, be premature at this stage to express a definite opinion for or against any degree of centralisation of refuse disposal, recent developments would seem to cast a doubt on the desirability of centralisation, as involving additional transport difficulties, and possibly other troubles from a health point of view. Further, it is impossible at present to say what, if any, land in a central position could be acquired by the Borough Councils for refuse disposal.

The final report, when submitted to the Conference when next it meets, may produce some scheme for the better and more expeditious treatment of the refuse of the Metropolis, and if this could be accomplished a real step forward would have been attained.

